# Supplementary material for: Development of chemokine network inhibitors using combinatorial saturation mutagenesis
Source: Commun Biol. 2025 Apr 3;8:549. doi: 10.1038/s42003-025-07778-6 (PMC11969024; doi:10.1038/s42003-025-07778-6)
Supplement: Supplementary file 4 — Supplementary Data 2 [file 42003_2025_7778_MOESM4_ESM.zip › SupplementaryData3/scripts/xattune_ic50.tar!/./script/analysis/JK/JKExactPvalues.docx]

Supplementary Table: Exact P values for pIC50 comparisons to control

| Experiment components | Control | Number of biological replicates | P value |
| --- | --- | --- | --- |
| ATC,RA,HD2 | HD2 | 3 | NA |
| ATC,RA,HD2CM304 | HD2 | 3 | 3.06e-03 |
| ATC,RA,HD2CM307 | HD2 | 3 | 4.60e-01 |
| THP1,INS,HD2 | HD2 | 3 | NA |
| THP1,INS,HD2CM304 | HD2 | 3 | 2.41e-01 |
| THP1,INS,HD2CM307 | HD2 | 3 | 6.80e-02 |
| THP1,RA,HD2 | HD2 | 3 | NA |
| THP1,RA,HD2CM304 | HD2 | 3 | 5.15e-01 |
| THP1,RA,HD2CM307 | HD2 | 3 | 1.72e-01 |
| THP1,ATHERO,HD2 | HD2 | 3 | NA |
| THP1,ATHERO,HD2CM304 | HD2 | 3 | 8.91e-01 |
| THP1,ATHERO,HD2CM307 | HD2 | 3 | 2.19e-02 |
| ATC,INS,HD2 | HD2 | 3 | NA |
| ATC,INS,HD2CM304 | HD2 | 3 | 3.15e-02 |
| ATC,INS,HD2CM307 | HD2 | 3 | 6.11e-01 |
| ATC,ATHERO,HD2 | HD2 | 3 | NA |
| ATC,ATHERO,HD2CM304 | HD2 | 3 | 3.86e-02 |
| ATC,ATHERO,HD2CM307 | HD2 | 3 | 5.28e-01 |
